# Serum brain natriuretic peptide levels may be a useful marker for early diagnosis of cardiomyopathy secondary to neuroblastoma: A case report

**DOI:** 10.1002/ccr3.8738

**Published:** 2024-04-26

**Authors:** Natsumi Fujiyama, Osamu Matsuo, Takahiro Yamashita, Kensaku Kohrogi, Fumiya Miyamura, Tadashi Anan, Kimitoshi Nakamura

**Affiliations:** ^1^ Department of Pediatrics, Graduate School of Medical Sciences Kumamoto University Kumamoto Japan

**Keywords:** BNP, cardiomyopathy, catecholamines, infant, neuroblastoma

## Abstract

Cardiomyopathy is a rare but serious complication associated with neuroblastoma. The brain natriuretic peptide level led to a diagnosis of secondary dilated cardiomyopathy before the worsening of heart failure symptoms.

## Graphical abstract

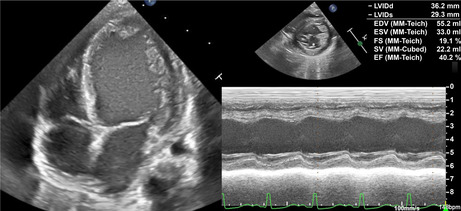

A 5‐month‐old boy with neuroblastoma showed dilated cardiomyopathy.

## 1 INTRODUCTION

Neuroblastoma is the most common neurogenic tumor in children that produces catecholamines and their metabolites. Neuroblastoma is the second most frequent solid cancer in children after cerebral tumors. It constitutes approximately 7% of all malignant tumors experienced during childhood.^1^ Although rare, there have been several reports of secondary cardiomyopathic complications due to excess catecholamines that may lead to severe heart failure symptoms.^2^, ^3^, ^4^, ^5^, ^6^, ^7^, ^8^

A few patients present with hypertrophic cardiomyopathy, of which dilated cardiomyopathy (DCM) is commonly observed.^9^, ^10^ According to previous reports, cardiomyopathy improves after neuroblastoma treatment.^7^, ^8^, ^9^, ^10^ We report the case of an infant with dilated cardiomyopathy complicated by neuroblastoma diagnosed by an abdominal mass, and early diagnosis was facilitated of cardiomyopathy by measuring serum brain natriuretic peptide (BNP) levels.

## 2 CASE HISTORY

**FIGURE 1:**
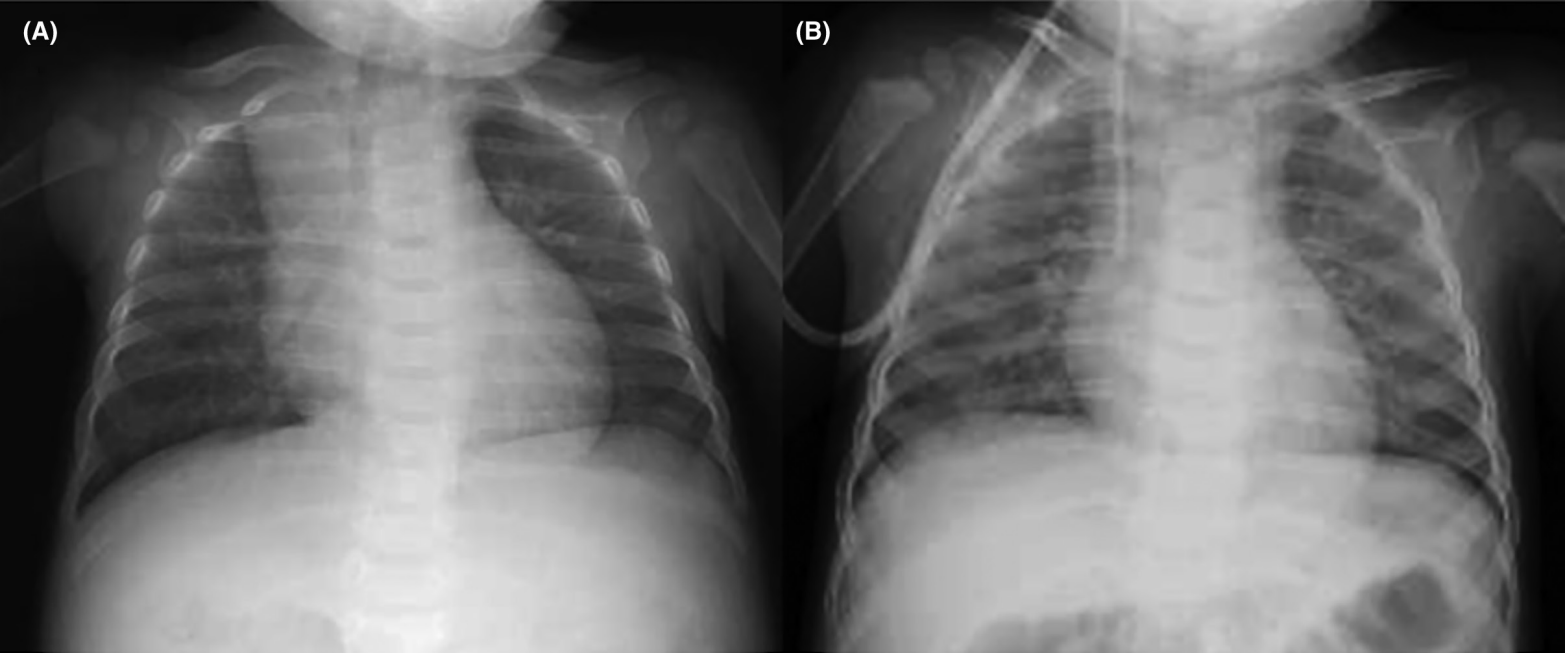
CTR improved as cardiomyopathy improved. (A) Before chemotherapy (CTR of 51%). (B) After the ninth course of chemotherapy (CTR of 44%) CTR, Cardio Thoracic Ratio.

**FIGURE 2:**
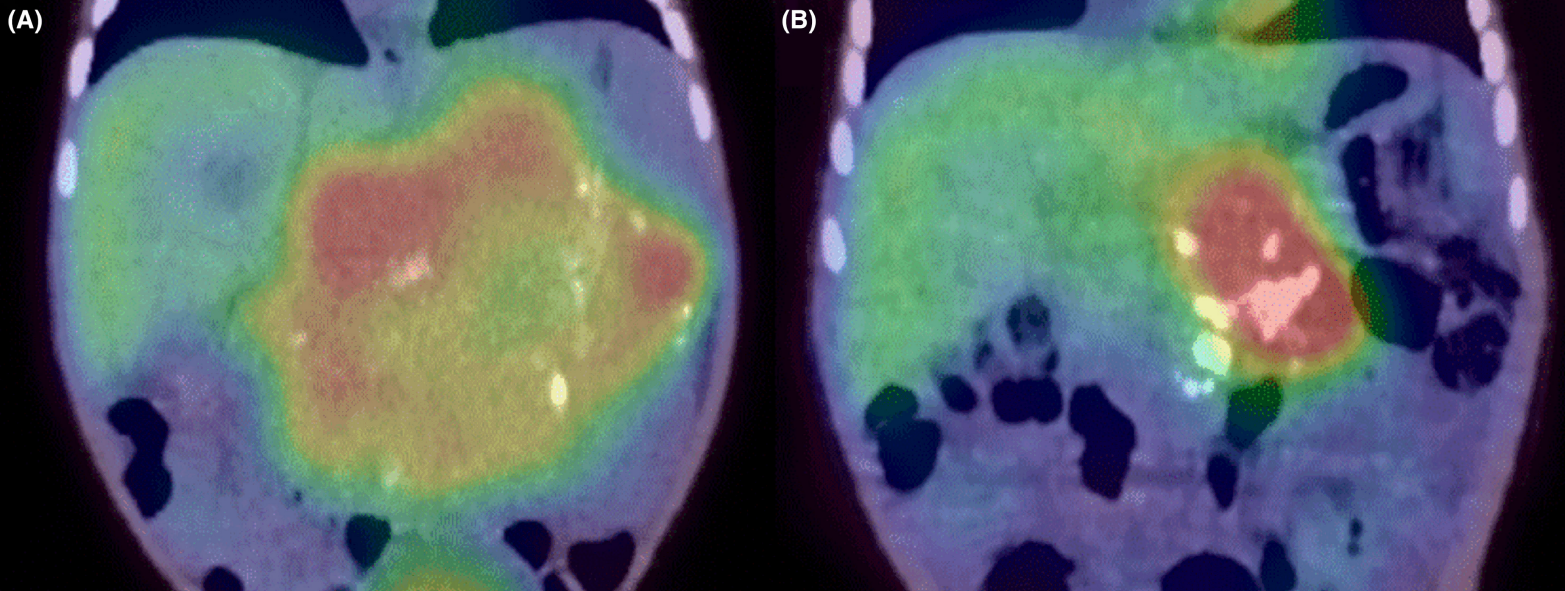
Chemotherapy was administered, and an MIBG scan showed that the tumor had shrunk (arrow). (A) Before chemotherapy. (B) After the ninth course of chemotherapy MIBG, metaiodobenzylguanidine.

**FIGURE 3:**
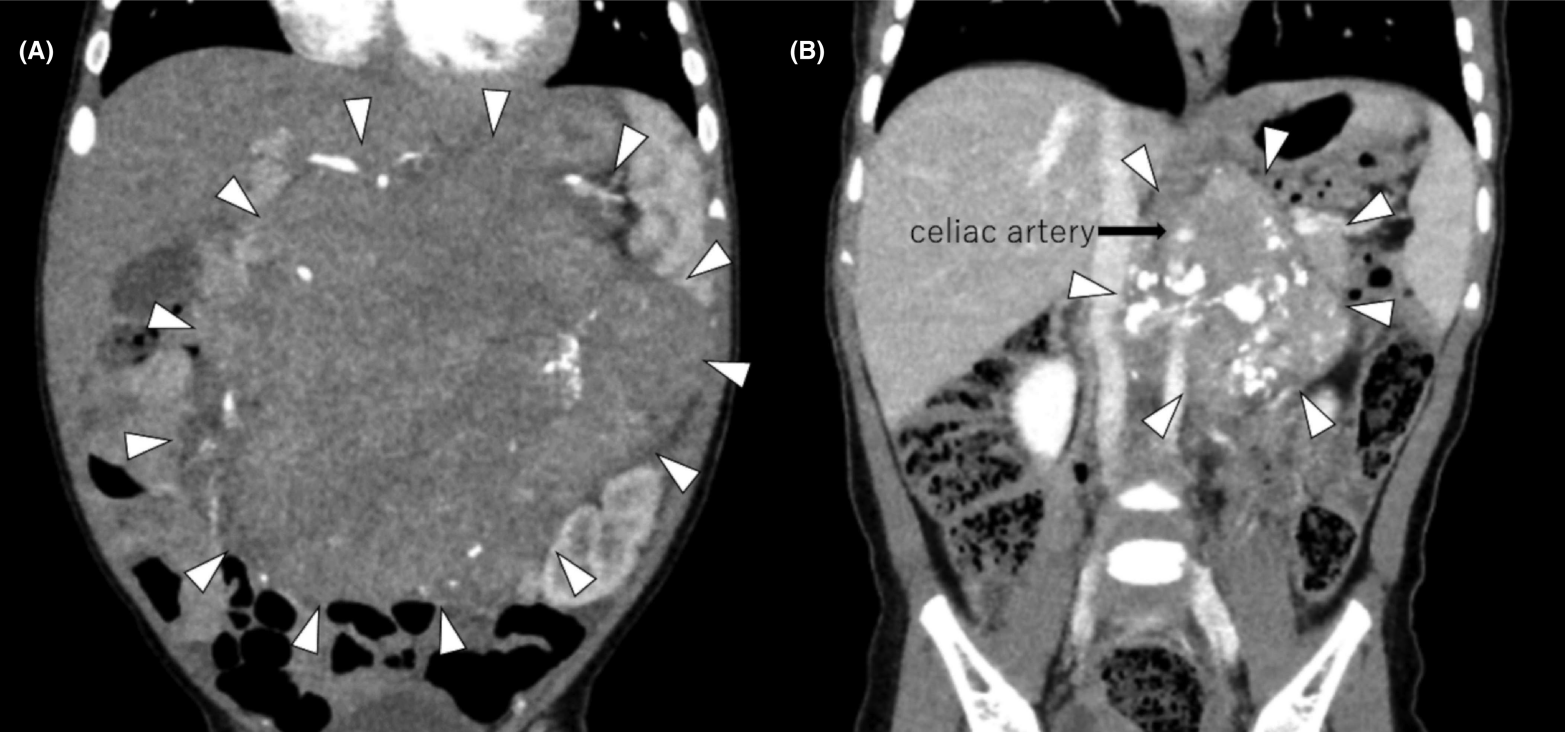
Chemotherapy was administered, and a CT scan showed the tumor (arrowhead) had shrunk with residual celiac artery involvement (arrow). (A) Before chemotherapy. (B) After the ninth course of chemotherapy. CT, computed tomography.

**FIGURE 4:**
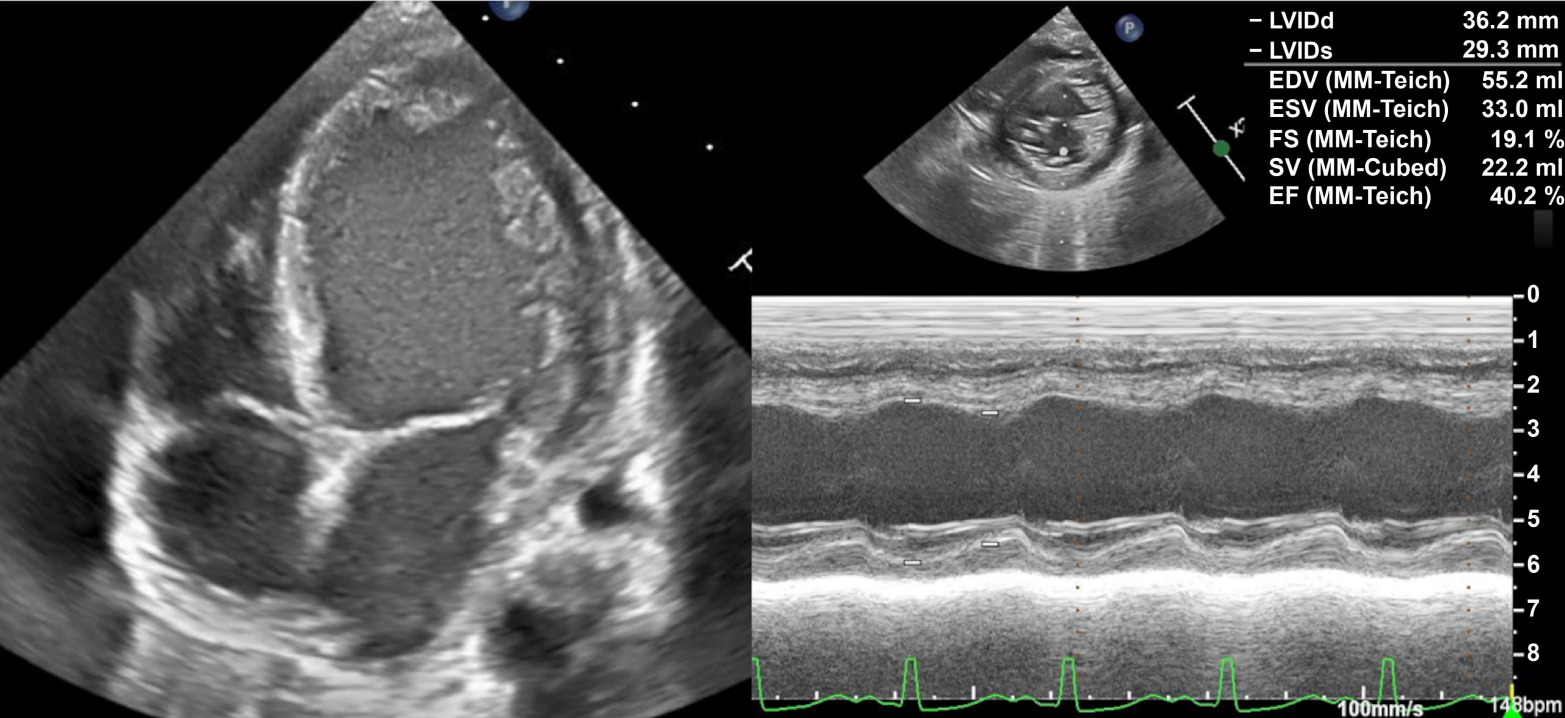
Admission echocardiography showed left ventricular enlargement and decreased contractility.

A 5‐month‐old boy with normal antenatal and perinatal history visited his general practitioner with a palpable abdominal mass. The patient was referred to our hospital with the suspicion of a solid tumor. Upon admission, the general condition of the patient was stable, indicated by a heart rate of 120 beats/min, a blood pressure of 98/50 mmHg, and a breathing rate of 35 breath/min. Chest x‐rays showed a slightly increased cardio thoracic ratio (CTR) of 51%, with no pulmonary congestion (Figure 1). The physical examination findings did not indicate congestive heart failure signs, such as peripheral coldness, pale complexion, and labored breathing. Neuron specific enolase (NSE), urine homovanillic acid (HVA), and vanilmandelic acid (VMA) levels were elevated, and I^123^‐ metaiodobenzylguanidine (MIBG) revealed an accumulation consistent with an abdominal mass (Figure 2), which increased the suspicion of neuroblastoma. There were no distant metastases. Contrast‐enhanced computed tomography (CT) of the abdomen presented a 10 × 8.7 × 9.0 cm mass in the retroperitoneum involving the celiac artery (Figure 3). However, there was no evidence of renal compression or entrapment of the renal artery. During the course of screening with blood tests, we observed elevated serum BNP levels. Furthermore, echocardiography showed dilatation of the left ventricle and a reduced left ventricular ejection fraction of 40% (Figure 4).

### 2.1 Investigations and treatment

**FIGURE 5:**
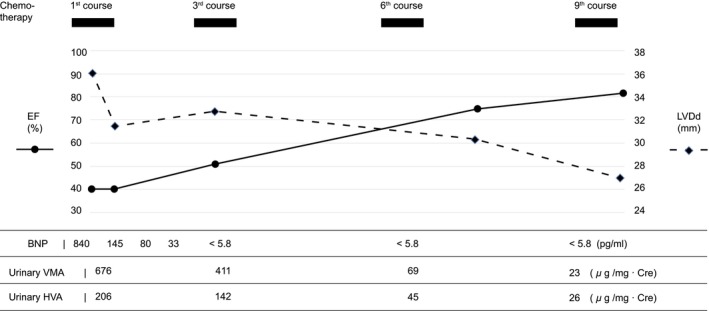
The course of treatment and changes in each data. After chemotherapy, urinary HVA and VMA improved at each evaluation. EF improved, and LVDd decreased in accordance with this improvement. BNP levels improved quickly with diuretics, milrinone, and landiolol hydrochloride. LVDd, left ventricular diastolic dimension; EF, ejection fraction; BNP, brain natriuretic peptide, HVA, homovanillic acid; VMA, vanilmandelic acid.

The patient was administered diuretics; however, the cardiac workload did not improve. Therefore, 0.3% milrinone 0.3γ and landiolol hydrochloride 4γ were added to the treatment regimen. Subsequently, the BNP improved (Figure 5). The disease course suggested cardiomyopathy caused by catecholamines produced by the tumor. In such cases, chemotherapy should be initiated immediately. The patient was stable enough to tolerate surgery, and a biopsy was performed. Pathological results confirmed the diagnosis of neuroblastoma. The patient was treated with chemotherapy with vincristine, cyclophosphamide, and teralbicin according to the Japanese Neuroblastoma Protocol, with The Children's Oncology Group (COG) risk classification as an intermediate‐risk county from the 30th day of admission. All three courses of chemotherapy, urinary VMA, HVA, contrast‐enhanced CT scan, and I^123^‐MIBG were performed. Evaluation after the third course revealed that the tumor had shrunk to a partial response; however, resection was impossible. Therefore, treatment was continued according to the protocol.

On the 40th day of admission, the patient remained stable without heart failure symptoms, and BNP normalized; milrinone, landiolol hydrochloride, and diuretics were tapered and switched from intravenous to oral therapy. The patient continued to have cardiac dysfunction due to cardiomyopathy; however, his BNP remained within the normal range. Periodic echocardiography disclosed improvement in the left ventricular dilatation and contractility after the ninth course of chemotherapy (Figure 5). Eventually, we continued administering carvedilol. However, furosemide and spironolactone were discontinued.

After the ninth course of chemotherapy, the tumor remained at 6 cm in diameter with residual celiac artery involvement, and total resection was considered difficult (Figures 2 and 3). After a second opinion, the family agreed to bring the patient for a follow‐up without additional treatment and to continue periodic outpatient evaluations for tumor markers, imaging, and cardiac function. Six months after discharge, the tumor shrank, cardiac function was normal, and carvedilol was discontinued.

## 3 DISCUSSION

Neuroblastoma is a catecholamine‐producing tumor that predominantly affects infants and is rarely associated with catecholamine cardiomyopathy. While most previously reported cases were diagnosed based on the progression of heart failure symptoms, our patient was diagnosed early because of an abdominal mass and elevated BNP levels.

Cardiomyopathy in catecholamine‐secreting tumors is caused by coronary and systemic vasoconstriction due to excessive catecholamine release, myocardial damage associated with tachycardia, free radical damage, downregulation of β‐adrenergic receptors, and sarcomere damage due to calcium influx, which may lead to hypertrophic cardiomyopathy (HCM), dilated cardiomyopathy (DCM), and Takotsubo cardiomyopathy.^11^

The most prevalent catecholamine‐producing tumor is pheochromocytoma. However, pheochromocytoma is less common in children, and neuroblastoma is more common.^11^ Pheochromocytomas often present with headaches, palpitations, hypertension, and myocardial hypertrophy, such as HCM. Takotsubo cardiomyopathy is often observed in pheochromocytoma‐associated myocardial diseases. However, DCM is associated with neuroblastoma, and our patient presented with DCM. A common feature of previously reported cases of cardiomyopathy is that patients were younger than 3 years of age. The sarcoplasmic reticulum (SR) and T‐tubules, which regulate Ca2+, an important medium for excitation‐contraction (E‐C) coupling, are small and immature in infants, making them susceptible to cardiac contractile dysfunction owing to catecholamine‐induced Ca2+ channel defects.

Consequently, infants are more susceptible to contractile cardiac dysfunction and dilated cardiomyopathies.^12^ In general, younger age is a favorable prognostic factor with regard to neuroblastoma. On the contrary, it is suggested that younger children may be more prone to complications of DCM for the above reasons.

Although urinary VMA and HVA levels, which reflect catecholamine production, are measured in catecholamine‐secreting tumors, urinary VMA and HVA levels do not predict myocardial damage as previously reported.^13^ Urinary VMA and HVA levels in neuroblastoma do not correlate with the stage and size of the neuroblastoma.^14^

Additionally, hypertension is not an indicator of cardiomyopathy, as a few previously reported cases of cardiomyopathy did not present with hypertension. In cases of hypertension associated with neuroblastoma, renal artery involvement and renal compression have been observed to a recognizable extent, suggesting a major involvement of the renin‐angiotensin‐aldosterone system.^4^, ^7^, ^10^

Here, the BNP level at admission compelled us to perform echocardiography promptly and diagnose cardiomyopathy. The ventricles primarily secrete BNP in response to myocardial stretching. BNP is a useful biomarker for heart failure and has been established as a class 1 diagnostic marker in the diagnosis and management of cardiomyopathy.^15^, ^16^ It is a product of fetal myocardial genes and is considered to be a phenotype of a type of compensatory mechanism for myocardial damage. Furthermore, it may represent not only the effects of external mechanical cardiac stress but also the degree of damage to the myocardial tissue itself. While hypertension, urinary VMA, and HVA cannot be used as indicators of cardiomyopathy in the diagnosis of neuroblastoma, BNP measurement may be a convenient marker for early suspicion of catecholamine‐induced myocardial damage.

Neuroblastoma‐associated cardiomyopathy is a rare but serious complication. Early diagnosis and optimal management of cardiomyopathy are essential for the treatment of primary disease. Additionally, further case accumulation is desirable.

## 4 CONCLUSION

Serum BNP levels are useful as a screening test to identify cardiomyopathic complications that cannot be predicted by catecholamine production or hypertension. Neuroblastoma is a common solid tumor in children. Early diagnosis of cardiomyopathy by measuring serum BNP levels, especially when an abdominal tumor is suspected, may significantly contribute to the prognosis of neuroblastoma treatment.

## AUTHOR CONTRIBUTIONS

**Natsumi Fujiyama:** Conceptualization; data curation; investigation; validation; visualization; writing – original draft. **Osamu Matsuo:** Conceptualization; data curation; investigation; supervision; validation; visualization; writing – review and editing. **Takahiro Yamashita:** Data curation; investigation; validation; writing – review and editing. **Kensaku Kohrogi:** Data curation; investigation; validation; writing – review and editing. **Fumiya Miyamura:** Data curation; investigation; validation; writing – review and editing. **Tadashi Anan:** Supervision; writing – review and editing. **Kimitoshi Nakamura:** Supervision; writing – review and editing.

## FUNDING INFORMATION

This study did not receive any specific grants from funding agencies in the public, commercial, or non‐profit sectors.

## CONFLICT OF INTEREST STATEMENT

The authors have no conflicts of interest.

## CONSENT

Written informed consent was obtained from the parents of the patient to publish this report, in accordance with the journal's patient consent policy.

## Data Availability

Data sharing is not applicable to this article as no datasets were generated or analyzed during the current study.
